# Curing Benign Paroxysmal Positional Vertigo (BPPV) Through Telehealth: A Case Series

**DOI:** 10.7759/cureus.16363

**Published:** 2021-07-13

**Authors:** Khalid Bashir, Abdulla Yousuf, Lubna Rauf, Mohamed Dewji, Amr Elmoheen

**Affiliations:** 1 Medicine, Qatar University, Doha, QAT; 2 Emergency Medicine, Hamad Medical Corporation, Doha, QAT; 3 Medical Education and Simulation, Hamad General Hospital, Doha, QAT; 4 Clinical Education, Qatar College of Medicine, Doha, QAT; 5 Medical Education, Primary Health Care Corporation, Doha, QAT

**Keywords:** benign paroxysmal positional vertigo, bppv, telehealth, covid-19, telemedicine, video telemedicine

## Abstract

Benign paroxysmal positional vertigo (BPPV) is a common medical condition in which the patient feels a spinning sensation when making certain head movements. There is evidence to support those free-floating calcium crystals in the semi-circular canals (the inner ear) may be the cause. BPPV can be a disabling condition. It can be easily diagnosed after taking a careful history and performing bedside examinations. BPPV can be treated successfully through a specific set of physical maneuvers leading to the removal of these crystals from the inner ear. We present three cases of BPPV, living in three different countries, treated successfully using telehealth via Zoom. This approach can be a particularly useful consultation stand during the coronavirus disease 2019 (COVID-19) pandemic.

## Introduction

Dizziness is a common medical condition. In the United States, over 90-million people are affected by dizziness annually. The condition appears to be more common in patients over the age of 75 [1]. Dizziness is often a loosely used common term that describes a group of sensations, including vertigo, disequilibrium, and presyncope. Each sensation has numerous etiologies. Most physicians can find it difficult to elucidate the extent of dizziness that a patient is experiencing, and some physicians find it difficult to assess the patient with dizziness properly and offer the physical maneuvers.

The vestibular system is categorized into central and peripheral components. The peripheral components comprise three semi-circular canals and the otolithic organs (saccule and utricle). The semi-circular canals detect the head’s rotational movement while the saccule and utricle respond to gravity and linear acceleration, respectively. The vestibular organs are constantly in a state of symmetrically tonic activity that stimulates the central vestibular system when excited. The central vestibular system continually processes this information and helps us maintain our sense of position and balance.

BPPV is one of the commonest peripheral vestibular disorders. At least 64 in every 100,000 Americans is affected [1]. The condition is more prevalent in women than in men, and symptoms usually present in the fourth and fifth decades of life. Epley proposed in 1980 that canaliths (free-floating densities) present in the semi-circular canals deflect the cupula, causing the vertigo sensation [1]. This has been documented in the Canalithiasis theory. There are three semicircular circular canals: posterior, lateral, and anterior. The posterior semicircular canal is commonly affected in almost 2/3rd of the cases [1].

BPPV patients often complain of vertigo after making changes in their head position, getting out of bed, or rolling over. Vertigo usually occurs suddenly and usually subsides within a minute in most cases. Remissions separate most attacks; however, patients may experience constant lightheadedness between episodes. Classic BPPV involves the posterior semi-circular canal and is characterized by geotropic nystagmus with a duration of fewer than 20 seconds.

Within the present context, coronavirus disease 2019 (COVID-19) is a novel disease that has created many challenges among clinicians due to its rapidly evolving nature. Dizziness or vertigo is one of the clinical symptoms of COVID-19. Several studies across the world have shown that dizziness is a major clinical manifestation of COVID-19 [2]. This may be important, considering that dizziness has been postulated with a possible viral infection.

A study in China found dizziness to be a major neurological manifestation of COVID-19 [3]. The occurrence of dizziness ensued the neuroinvasive potential of the COVID-19 virus. According to Baig et al., the virus permeates the neural tissue from the circulation and attaches itself to the receptors of the angiotensin-converting enzyme 2 present in the capillary endothelium [3]. Also, direct invasion, hypercoagulopathy, hypoxia, and immune-mediated insult were postulated as mechanisms of neuroinvasion that caused dizziness [4]. The aim of this case series is to assess whether BPPV can be treated ever more clearly using digital platforms such as Zoom. All three patients did not suffer from COVID-19 infection, hence their vertigo was of primary origin, with no known cause.

## Case presentation

Case 1

A 58-year-old physician resident in Egypt contacted an expert based in Qatar. She had been suffering from recurrent vertigo for almost four months. There was no other significant past medical history, never diagnosed as COVID-19, and was not currently taking any medications. The consultation was done online through Zoom. The phone camera in the patient’s house was held close to her eyes and face by a family member to allow for a closer examination. She was diagnosed with left-sided posterior canal BPPV. She was treated with an Epley maneuver with a successful outcome. Symptoms completely settled, and there was no recurrence of symptoms three months after the maneuver.

Case 2

A 38-year-old female patient resident in Hungary had a telehealth consultation for vertigo lasting for almost eight months. She had no other significant past medical history or diagnosis as COVID-19 before. Her husband maneuvered the mobile phone camera to allow for a close examination, and she was diagnosed with posterior canal BPPV of the right ear. She was treated with a modified Epley maneuver with a successful outcome. She reported no recurrence of symptoms three months after the manure.

Case 3

**Figure 1:**
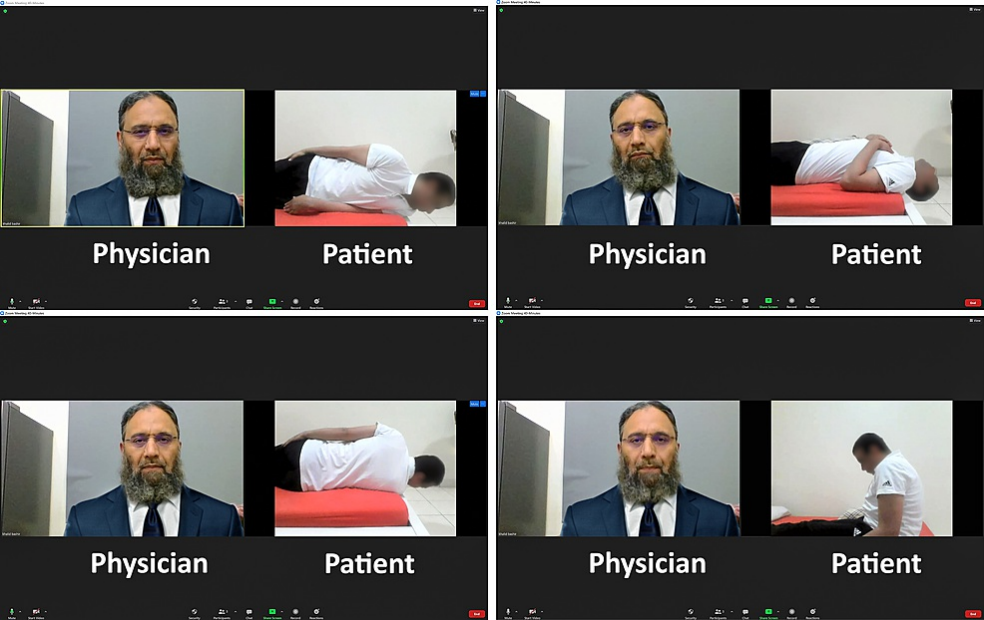
A computer screenshot showing an online consultation on Zoom for treating BPPV BPPV, Benign Paroxysmal Positional Vertigo

A 52-year-old gentleman resident in Pakistan contacted the physician with vertigo symptoms of seven months duration. His other previous significant history included controlled hypertension. He was not diagnosed as COVID-19. At his Zoom consultation (Figure 1), he was diagnosed with has right-sided posterior canal BPPV, again facilitated by a close camera view of his eyes. He was treated with the modified Epley maneuver with a successful outcome. There was no recurrence of his symptoms three months post maneuver.

## Discussion

COVID-19 has severely affected healthcare globally in many ways [5]. The healthcare industry is particularly concerned about the overstretch of healthcare facilities [6]. There is a significant challenge facing the provision of primary healthcare at this time, as services are continually disrupted due to lockdown, shortage of protective gear, and the risk of spreading infection [7]. To mitigate and manage the spread of COVID-19, healthcare providers can boost their systems’ efficiency by utilizing digital technologies in their medical systems [8].

As such, many physicians are providing remote care via telemedicine services [9]. These telemedicine services provide several non-dispensing functions, thus allowing the clinician to deliver quality medical consultations during the pandemic. Among such services are health education, reviewing the patient’s medical histories, reviewing drug use, imitated examinations, and health therapy management [9].

Telemedicine care can be incorporated into the medical operations systems to maximize the efficient delivery of healthcare [5,10]. It is key to promoting social distancing and assisting medical facilities in curbing the incidence of prolonged waiting times [11]. By reducing physical contact among clinicians and patients, and in-person visits, virtual medical care solutions can help curb the spread of the virus and protect healthcare staff from infection [12].

COVID-19-related otoneurological symptoms, like balance disorders and tinnitus, are common [13-14]. It is important to note that neuroinvasive and neurotrophic capabilities are typical of several coronaviruses [15]. COVID-19 effects on the neuronal tissue may be due to an infection of the central nervous system or due to damage of the vascular tissues via vasculopathy or vasculitis [16]. Recently in the literature, cases reports have been published where patients developed BPPV after COVID-19 infection. Hence, it is important that patients who have dizziness after COVID-19 infection should be assessed for BPPV, and potentially curative treatment can be provided [17].

Balance disorders may depend on the damage to the vascular system due to the high susceptibility of the inner ear structures to ischemia vis their high energy requirements and features of their terminal vasculature. Primary and secondary vasculature are linked to audiovestibular symptoms. Dizziness may also indicate primary cardiovascular disease [18-19]. Viral infections, such as hepatitis B and C, may be linked with vasculitis. Studies have shown that vasculitis is a major clinical indication of COVID-19 [20]. Also, benign paroxysmal positional vertigo is indicated in COVID-19 cases, although there is no published data to this effect.

## Conclusions

Telemedicine has been used in the provision of healthcare for a long time. It has been used to link with rural communities and deliver remote healthcare to patients. This has usually yielded excellent results. Tele healthcare is widely available and more so with increased access to high-speed internet, cost-effective, and readily accepted by patients and clinicians. With the outbreak of the COVID-19 pandemic, the utilization of telemedicine by patients and clinicians has become an absolute necessity.

This article has examined three cases of BPPV successfully treated with telehealth. It could be hypothesized that otoneurological symptoms, such as balance disorders, may present in COVID-19 patients due to multifactorial and can be successfully treated either pharmacologically or via telehealth to prevent long-term disability. The primary risk of using this consulting portal is the small possibility of missing a rare intracranial pathology.
